# Anti-Osteoarthritic Effects of Antarctic Krill Oil in Primary Chondrocytes and a Surgical Rat Model of Knee Osteoarthritis

**DOI:** 10.3390/md21100513

**Published:** 2023-09-28

**Authors:** Sae-Kwang Ku, Jong-Kyu Kim, Yoon-Seok Chun, Chang-Hyun Song

**Affiliations:** 1 Department of Anatomy and Histology, College of Korean Medicine, Daegu Haany University, Gyeongsan 38610, Republic of Korea; gucci200@dhu.ac.kr; 2 AriBnC Co., Ltd., Yongin 16914, Republic of Korea; swrhrnak@gmail.com (J.-K.K.); ceochun@aribnc.com (Y.-S.C.)

**Keywords:** OA, cartilage, marine, PUFA, anti-inflammation, MMP, anti-apoptosis, chondrogenesis

## Abstract

Osteoarthritis (OA) is characterized by progressive cartilage destruction and synovitis; however, there are no approved disease-modifying OA drugs. Krill oil (KO) has been reported to possess anti-inflammatory properties and alleviate joint pain in knee OA, indicating its potential to target the inflammatory mechanism of OA. Therefore, the anti-OA effects of KO were investigated in primary chondrocytes and a surgical rat model of knee OA. The oral administration of KO at 200 and 100 mg/kg for 8 weeks improved joint swelling and mobility in the animal model and led to increased bone mineral density and compressive strength in the cartilage. The oral KO doses upregulated chondrogenic genes (*type 2 collagen*, *aggrecan*, and *Sox9*), with inhibition of inflammation markers (5-lipoxygenase and prostaglandin E_2_) and extracellular matrix (ECM)-degrading enzymes (MMP-2 and MMP-9) in the cartilage and synovium. Consistently, KO treatments increased the viability of chondrocytes exposed to interleukin 1α, accompanied by the upregulation of the chondrogenic genes and the inhibition of the ECM-degrading enzymes. Furthermore, KO demonstrated inhibitory effects on lipopolysaccharide-induced chondrocyte inflammation. Histopathological and immunohistochemical analyses revealed that KO improved joint destruction and synovial inflammation, probably due to the anti-inflammatory, anti-apoptotic, and chondrogenic effects. These findings suggest the therapeutic potential of KO for knee OA.

## 1. Introduction

Osteoarthritis (OA), known as a degenerative joint disease, is the most prevalent type of arthritis among the elderly, impacting over 7% of the global population [1]. The knee, being a pivotal weight-bearing joint, is notably vulnerable to OA, with knee OA prevalence accounting for more than 80% of all OA cases [2]. Beyond aging, primary risk factors for developing knee OA include genetics, female gender, obesity, metabolic alterations, traumatic joint injuries, and muscle weakness [3]. Knee OA is considered a whole joint disease involving all joint tissues, including a progressive degeneration of cartilage, subchondral bone remodeling, inflammation and fibrosis of synovial membrane, meniscal degeneration, and inflammation and fibrosis of the infrapatellar fat pads [4,5]. The underlying pathogenesis involves the gradual breakdown of articular cartilage due to chondrocyte death and degradation of the extracellular matrix (ECM), primarily composed of type 2 collagen (Col-2) and proteoglycans [3,6]. This disruption arises from an imbalance between anabolic and catabolic processes. The pathological articular cartilage eventually exhibits a significant reduction in its layer thickness and mechanical properties, such as stiffness, compared to healthy cartilage [5]. Moreover, maladaptive inflammatory pathways further exacerbate joint ECM degeneration, resulting in knee pain and stiffness, and physical impairments [7]. The societal and economic repercussions of knee OA are substantial, significantly compromising patients’ quality of life [1]. Despite the increasing prevalence associated with the rise in longevity and obesity, no approved disease-modifying drugs for OA are currently available. This underscores an urgent need for comprehensive therapeutic strategies aimed at preventing cartilage deterioration and promoting repair.

Primary treatments for knee OA are based on non-pharmacological and pharmacological measures [8,9]. Non-pharmacological options include weight loss, exercises, and physical therapy, while pharmacological recommendations involve the use of non-steroidal anti-inflammatory drugs (NSAIDs), acetaminophen, or corticosteroids. Although pharmacological treatments provide symptomatic relief, they cannot prevent cartilage degradation or repair damaged cartilage. Furthermore, the long-term use of these medications yields adverse effects, such as cardiovascular and gastrointestinal complications, and renal failure [10,11]. Certain NSAIDs even impede cartilage function by inhibiting proteoglycan synthesis in the ECM [12]. Glucosamine and chondroitin sulfate, which are essential substrates for proteoglycan biosynthesis, are widely used dietary supplements for chondroprotection in OA. Nevertheless, they have not been approved by the regulatory bodies for knee OA treatments, and their effectiveness remains controversial [13,14]. Patients suffering from chronic knee pain and disability often try to consume various nutritional supplements derived from natural products (i.e., curcumin, green tea, resveratrol, citrus fruit, etc.) to enhance joint health or alleviate OA symptoms [15].

Recently, there has been a growing interest in the development of disease-modifying OA drugs aimed at targeting pathways related to joint tissue degeneration [16]. Obesity not only increases the mechanical load on the knee joint but also appears to be a predisposing factor for OA in terms of low-grade systemic inflammation and altered lipid metabolism of free fatty acids (FAs) [17]. Polyunsaturated FAs (PUFAs) act as precursors to potent lipid mediators known as eicosanoids. Eicosanoids derived from omega-3 (n-3) and n-6 PUFAs exhibit opposing effects on inflammatory responses, with anti-inflammatory and proinflammatory properties, respectively [18]. The n-3/n-6 PUFA ratio has been observed to be lower in patients with knee OA, suggesting that maintaining the dominant ratio through dietary supplementation with n-3 PUFA could play a crucial role in inhibiting OA progression [19]. The major sources of n-3 PUFAs, especially long-chain n-3 PUFAs including eicosapentaenoic acid (EPA) and docosahexaenoic acid (DHA), are derived from fish and other marine organisms [20]. Notably, EPA and DHA, the n-3 PUFAs have shown anti-inflammatory and cartilage-protective effects [21], demonstrating significant potential as natural anti-inflammatory agents in experimental OA models [22,23,24]. It has been reported that dietary supplementation with n-3 PUFAs alleviates OA symptoms, while n-6 PUFAs increase the risk of OA development [25,26].

Krill oil (KO), extracted from Antarctic krill (*Euphausia superba*), is abundant in long-chain n-3 PUFAs in the form of phospholipids, primarily composed of EPA and DHA [27,28]. KO also has potent antioxidants, astaxanthin, and flavonoids, known to suppress inflammation [29]. Previous randomized controlled trials have reported that dietary KO reduces subjective arthritic symptoms and systemic inflammation in patients with chronic inflammation including OA [30], and improves joint pain and stiffness in patients with mild to moderate knee OA [31,32]. Dietary supplementation of KO has shown beneficial effects through its anti-inflammatory properties in animal models of monosodium iodoacetate (MIA)-induced knee OA [33], rheumatoid arthritis [34], low-grade inflammation [35], and obesity [36,37]. Moreover, the oral administration of KO ameliorates joint cartilage degeneration by activating chondrocyte autophagy and inhibiting apoptosis in a surgical mouse model of knee OA [38]. Our previous study demonstrated the protective effects of KO on metabolic syndromes via anti-inflammation, antioxidation, and anti-apoptosis in a high-fat diet-induced obese mice model [39]. Therefore, the anti-OA effects of KO and the relevant mechanisms were examined in primary chondrocytes and a surgical rat model of knee OA.

## 2. Results

### 2.1. Chondroprotective Effects In Vitro

**Figure 1:**
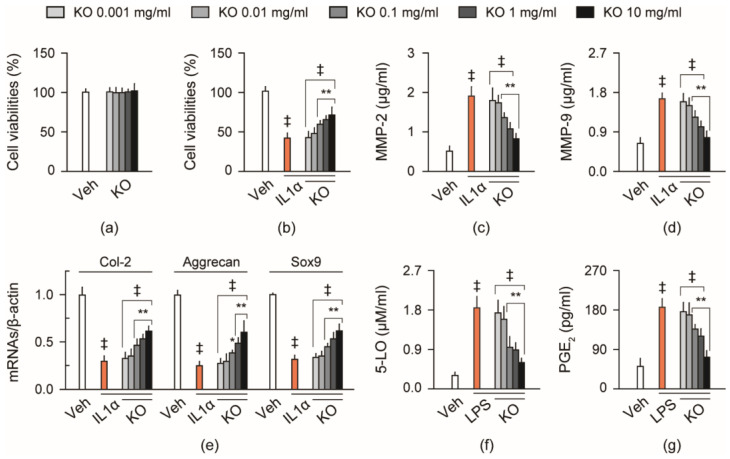
Cytoprotective effects in vitro. (**a**) Cell viability in primary articular chondrocytes. (**b**) Cell viability in the chondrocytes exposed to interleukin (IL)1α. (**c**,**d**) Levels of metalloproteinase (MMP)-2 and MMP-9 in the exposure to IL1α. (**e**) Relative expressions of chondrogenic genes, *type 2 collagen* (*Col-2*), *aggrecan*, and *sex-determining region Y-box 9* (*Sox9*), in the exposure to IL1α. (**f**,**g**) 5-lipoxygenase (LO) activity and prostaglandin E2 (PGE2) level in chondrocytes exposed to lipopolysaccharide (LPS). Values are expressed as the means ± standard deviations (SDs) in six independent experiments after treatments of krill oil (KO) at the indicated doses. ‡: *p* < 0.01, compared to the vehicle-treated normal control (Veh); **: *p* < 0.01 and *: *p* < 0.05, compared to the vehicle-treated negative control exposed to IL1α (IL1α) or LPS (LPS).

Rat primary chondrocytes were isolated from the knee joint and treated with KO at five doses of 0.001 to 10 mg/mL in distilled water (DW) as a vehicle for 24 h (Figure 1). Cell viability was not significantly different in treatments of KO at all doses compared to the vehicle-treated normal control (Figure 1a). In the interleukin (IL)1α-induced cell injury model, cell viability was reduced in the vehicle-treated IL1α model control compared to the normal control (*p* < 0.01, Figure 1b). However, it was significantly increased in the treatments of KO at 0.1, 1, and 10 mg/mL compared to the IL1α model control (*p* < 0.01). The effective concentration producing the half-maximal response (EC_50_) was calculated as 68.5 μg/mL. Additionally, in the IL1α model control compared to the normal control, the levels of matrix metalloproteinase (MMP)-2 and MMP-9 were increased (*p* < 0.01, Figure 1c,d), and the expressions of chondrogenic genes, *Col-2*, *aggrecan*, and *sex-determining region Y-box 9* (*Sox9*), were reduced (*p* < 0.01, Figure 1e). However, the levels of MMP-2 and MMP-9 were significantly reduced in treatments of KO at 0.1, 1, and 10 mg/mL compared to the IL1α model control (*p* < 0.01), and the gene expressions of Col-2, aggrecan, and Sox9 were increased (*p* < 0.05). In the lipopolysaccharides (LPS)-induced cell inflammation model, 5-lipoxygenase (5-LO) activity and prostaglandin E (PGE)2 level were increased in the vehicle-treated LPS model control compared to the normal control (*p* < 0.01); however, they were reduced in treatments of KO at 0.1, 1, and 10 mg/mL compared to the LPS model control (*p* < 0.01, Figure 1f,g).

### 2.2. Improvements in Knee Joint Thickness In Vivo

**Figure 2:**
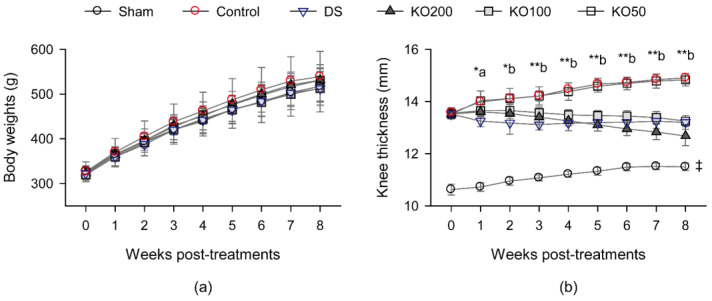
Changes in body weight and knee thickness in vivo. (**a**) Body weight. (**b**) Knee thickness. Values are expressed as the means ± SDs in eight sample sizes. ‡: *p* < 0.01, compared to the sham; a *: *p* < 0.05, in the DS and KO200 groups compared to the osteoarthritis rat model control (control); b **: *p* < 0.01 and b *: *p* < 0.05, in the DS, KO200, and KO100 groups compared to the control.

Sham and one of the knee OA rat model groups were orally administered with DW as the normal and OA controls, respectively. The other OA model groups were orally administered with diclofenac sodium at 2 mg/kg (DS) or KO at 200, 100, and 50 mg/kg (KO200, KO100, and KO50, respectively). The kinetic changes in body weight and knee thickness were measured every week for 8 weeks post treatment (Figure 2). There were no differences in the body weight changes among the groups (Figure 2a). However, two-way analysis of variance (ANOVA) for the joint thickness showed significant main effects for the groups and timepoints measured (*p* < 0.01, Figure 2b). There were also significant interactions between the groups and timepoints (*p* < 0.01), indicating the timepoint-dependent differences in the joint thickness among the groups. Post hoc tests revealed significant increases in the knee thickness in the OA model control compared to the sham (*p* < 0.01). However, compared to the OA control, the knee thickness was significantly reduced in the DS and KO200 groups during weeks 1–8 post treatment and in the KO100 during weeks 2–8 (*p* < 0.05). Furthermore, the thickness was reduced more in the KO200 than in the DS during weeks 7–8 (*p* < 0.05).

### 2.3. Improvements in Knee Joint Extension In Vivo

**Figure 3:**
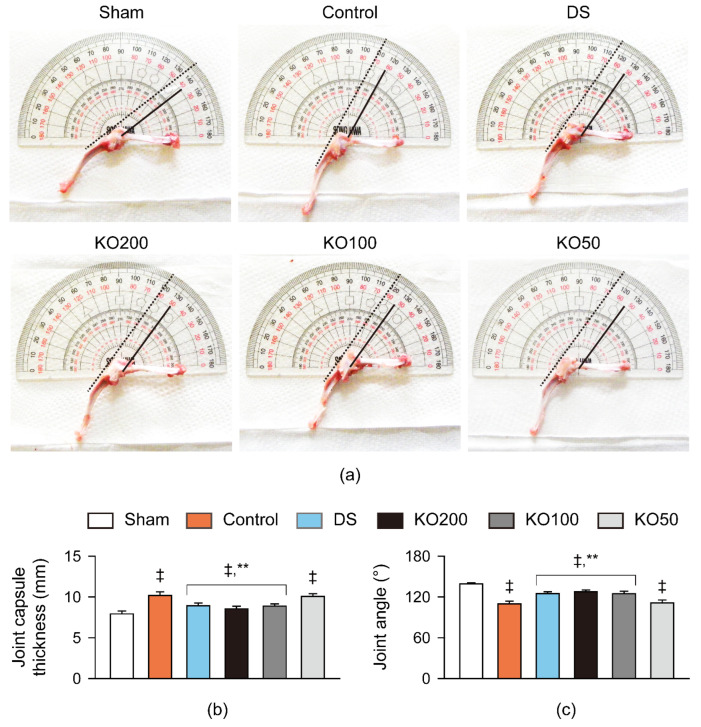
Joint capsule thickness and extension angle in vivo. (**a**) Representative images for the joint capsule thickness and extension angle. Lines and dotted lines indicate the measured angle and guiding lines for the measurements, respectively. (**b**) Thickness of the joint capsule. (**c**) Maximal joint extension angle. Values are expressed as the means ± SDs in eight sample sizes. ‡: *p* < 0.01, compared to the sham; **: *p* < 0.01, compared to the control.

Following all treatments, the thickness of the knee joint capsule exposed from the surrounding tissues was directly measured, and the maximum extension angle of the joint was assessed (Figure 3). Joint capsule swelling and stiffness were evident in the OA control, while they were mild in the DS and KO treatment groups (Figure 3a). Indeed, the thickness of the joint capsule was increased in the OA control compared to the sham (*p* < 0.01); however, it was significantly reduced in the DS, KO200, and KO100 groups compared to the OA control (*p* < 0.01, Figure 3b). The maximal extension angle was reduced in the OA control compared to the sham (*p* < 0.01); however, it was increased in the DS, KO200, and KO100 compared to the OA control (*p* < 0.01, Figure 3c).

### 2.4. Improvements in Bone Mineral Deposition and Compressive Strength in Joint Cartilage In Vivo

**Figure 4:**
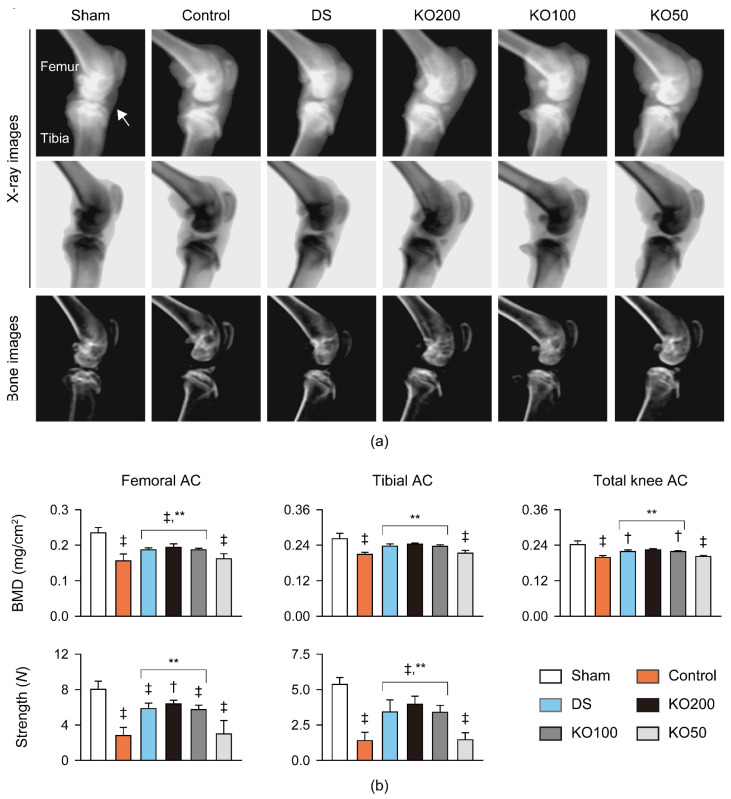
Bone mineral density (BMD) and compressive strength in vivo. (**a**) Representative images for joint tissues in live dual-energy X-ray absorptiometry. An arrow indicates joint capsule. (**b**) BMD and compressive strength in the femoral and tibial articular cartilage (AC). Values are expressed as the means ± SDs in eight sample sizes. ‡: *p* < 0.01 and †: *p* < 0.05, compared to the sham; **: *p* < 0.01, compared to the control.

Bone mineral density (BMD) was analyzed in the articular cartilages using live dual-energy X-ray absorptiometry (DEXA) images, and the comprehensive strength was measured (Figure 4). In DEXA images, joint capsule swelling, erosive cartilage degradation, and osteophyte formation were severe in the OA model control; however, they were attenuated in the DS and KO treatment groups (Figure 4a). In the total and each of the femoral and tibial articular cartilages, BMD was reduced in the OA control compared to the sham (*p* < 0.01); however, it was increased in the DS, KO200, and KO100, compared to the OA control (*p* < 0.01, Figure 4b). The comprehensive strength of both femoral and tibial articular cartilages was reduced in the OA control compared to the sham (*p* < 0.01); however, it was increased in the DS, KO200, and KO100, compared to the OA control (*p* < 0.01).

### 2.5. Inhibition of Inflammation and ECM Degradation in Joint Tissue In Vivo

**Figure 5:**
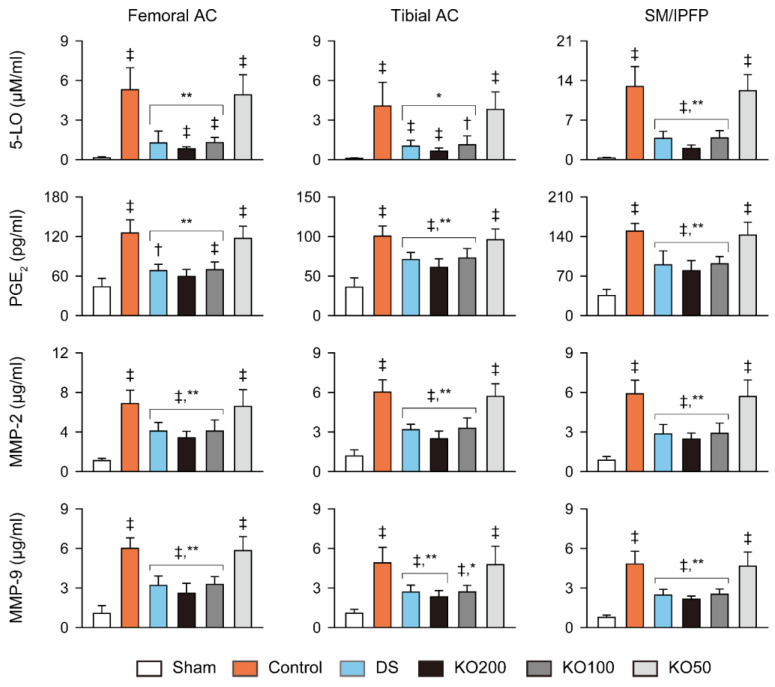
Anti-inflammatory and anti-extracellular (ECM) degradation activities in vivo. Activity of 5-lipoxygenase (5-LO) and levels of prostaglandin E (PGE)_2_, matrix metalloproteinase (MMP)-2, and MMP-9 in the femoral and tibial AC and the synovial membrane/infrapatellar fat pad (SM/IPFP). Values are expressed as the means ± SDs in eight sample sizes. ‡: *p* < 0.01 and †: *p* < 0.05, compared to the sham; **: *p* < 0.01 and *: *p* < 0.05, compared to the control.

The activities of 5-LO and levels of PGE_2_, MMP-2, and MMP-9 in the femoral and tibial articular cartilages and the synovial membranes including infrapatellar fat pads, were increased in the OA control compared to the sham (*p* < 0.01); however, they were significantly reduced in the DS, KO200, and KO100 compared to those of the OA control (*p* < 0.05, Figure 5). In particular, the 5-LO activity in the synovial membrane/infrapatellar fat pad was reduced more in the KO200 than in the DS (*p* < 0.05).

### 2.6. Upregulation of Chondrogenic Genes in Joint Tissue In Vivo

**Figure 6:**
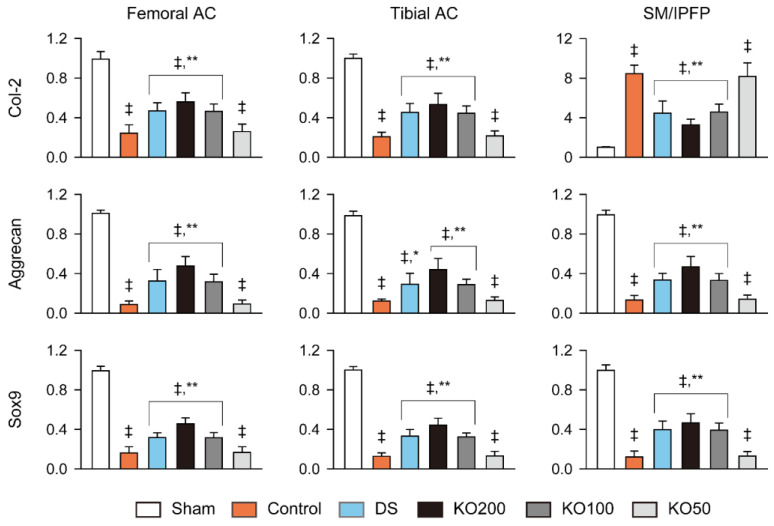
Gene expressions of Col-2, aggrecan, and Sox9 in the femoral and tibial AC and the SM/IPFP. Values are expressed as the means ± SDs in eight sample sizes. ‡: *p* < 0.01, compared to the sham; **: *p* < 0.01 and *: *p* < 0.05, compared to the control.

Chondrogenic genes of *Col-2*, *aggrecan*, and *Sox9* in the femoral and tibial articular cartilages and the synovial membranes including infrapatellar fat pads, were downregulated in the OA control compared to the sham (*p* < 0.01); however, they were significantly upregulated in the DS, KO200, and KO100 compared to the OA control (*p* < 0.05, Figure 6). In the synovial membrane/infrapatellar fat pad, the *aggrecan* and *Sox9* were downregulated in the OA control compared to the sham (*p* < 0.01), while the *Col-2* was upregulated (*p* < 0.01). However, the gene expressions were significantly reversed in the DS, KO200, and KO100 compared to the OA control (*p* < 0.01). In the KO200 compared to the DS, the *Sox9* was upregulated more in both femoral and tibial cartilages, and the *aggrecan* was upregulated more in the femoral cartilage (*p* < 0.01).

### 2.7. Histopathological Improvements in Cartilage Destruction and Synovial Inflammation In Vivo

**Figure 7:**
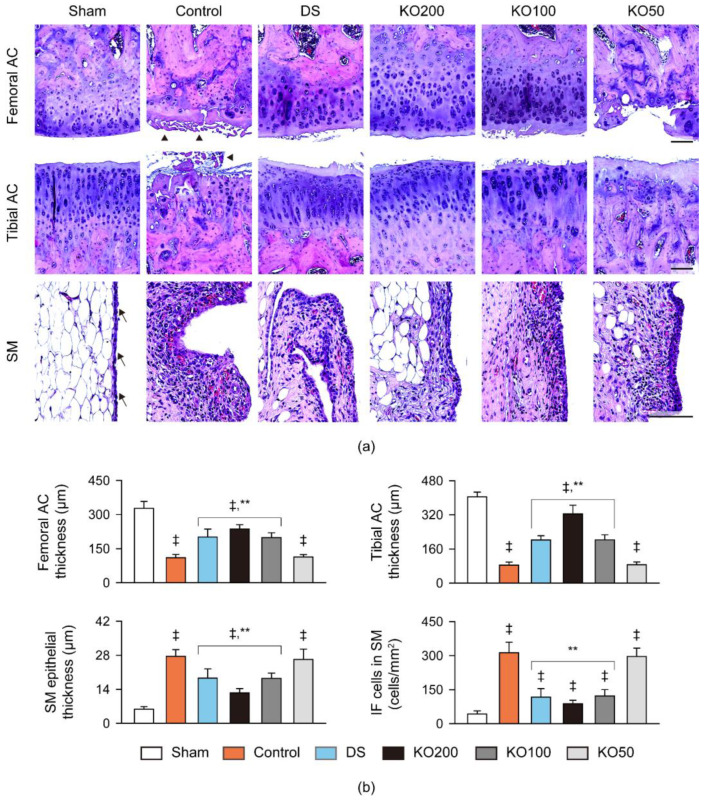
Histopathological analysis in vivo. (**a**) Representative images in the femoral and tibial AC and the SM, stained in hematoxylin and eosin. Arrowheads and arrows indicate the clone formation in the articular surfaces and SM-lining epithelium, respectively. Scale bars = 100 μm. (**b**) Thickness of both AC and the SM-lining epithelium and the number of infiltrated inflammatory (IF) cells in the SM. Values are expressed as the means ± SDs in eight sample sizes. ‡: *p* < 0.01, compared to the sham; **: *p* < 0.01, compared to the control.

**Figure 8:**
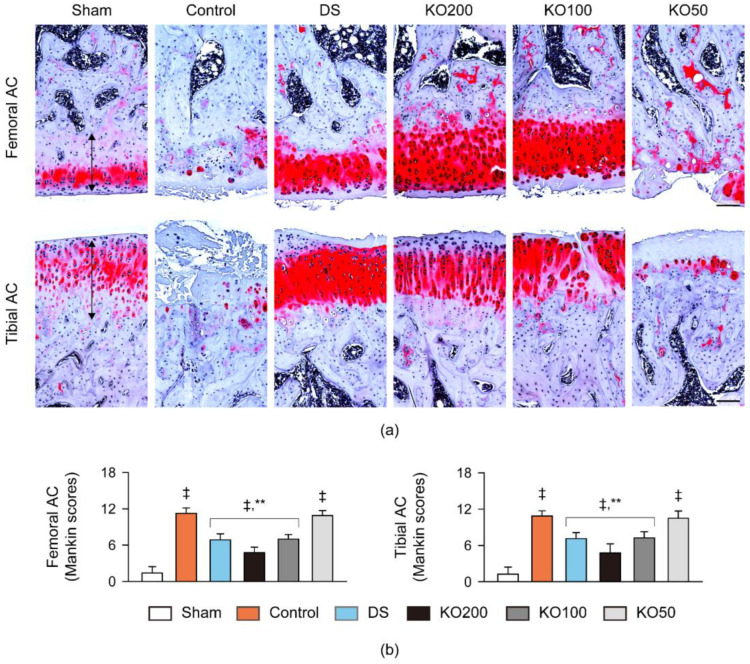
Histopathological scores in vivo. (**a**) Representative images in the femoral and tibial AC, stained with Safranin O. Double-ended arrows indicate AC thickness. Scale bars = 100 μm. (**b**) Mankin scores in both cartilages. Values are expressed as the means ± SDs in eight sample sizes. ‡: *p* < 0.01, compared to the sham; **: *p* < 0.01, compared to the control.

Histopathological changes were examined in the joint cartilages and synovial membrane/infrapatellar fat pad stained with hematoxylin and eosin (HE) and Safranin O (Figure 7 and Figure 8). In HE stains, thin cartilage layers and severe cartilage degradation were evident in the femoral and tibial articular surfaces of the OA control, along with thick synovial membrane-lining epithelium and the marked inflammatory cell infiltration (Figure 7a). However, the histopathological changes were attenuated in the DS and KO treatment groups. The thickness of the femoral and tibial articular cartilages was reduced in the OA control compared to the sham (*p* < 0.01); however, it was increased in the DS, KO200, and KO100 compared to the OA control (*p* < 0.01, Figure 7b). Conversely, the synovial membranes-lining epithelium and inflammatory cell infiltration were increased in the OA control compared to the sham (*p* < 0.01); however, they were reduced in the DS, KO200, and KO100 compared to the OA control (*p* < 0.01). Additionally, in the KO200 compared to the DS, the thickness of both cartilages was increased more, while the thickness of the synovial membrane-lining epithelium was reduced more (*p* < 0.05). In Safranin O stains, the OA control group exhibited evident cartilage injuries with reduced chondrocytes, clone formation, and stain intensity in the femoral and tibial surfaces; however, the changes were mild in the DS and KO treatment groups (Figure 8a). The Mankin scores in both articular cartilages were increased in the OA control compared to the sham (*p* < 0.01). However, the scores were significantly reduced in the DS, KO200, and KO100 compared to the OA control, and further reduced in the KO200 compared to the DS (*p* < 0.01, Figure 8b).

### 2.8. Immunohistochemistry for Anti-Inflammation, Anti-Apoptosis, and Chondrogenesis In Vivo

**Figure 9:**
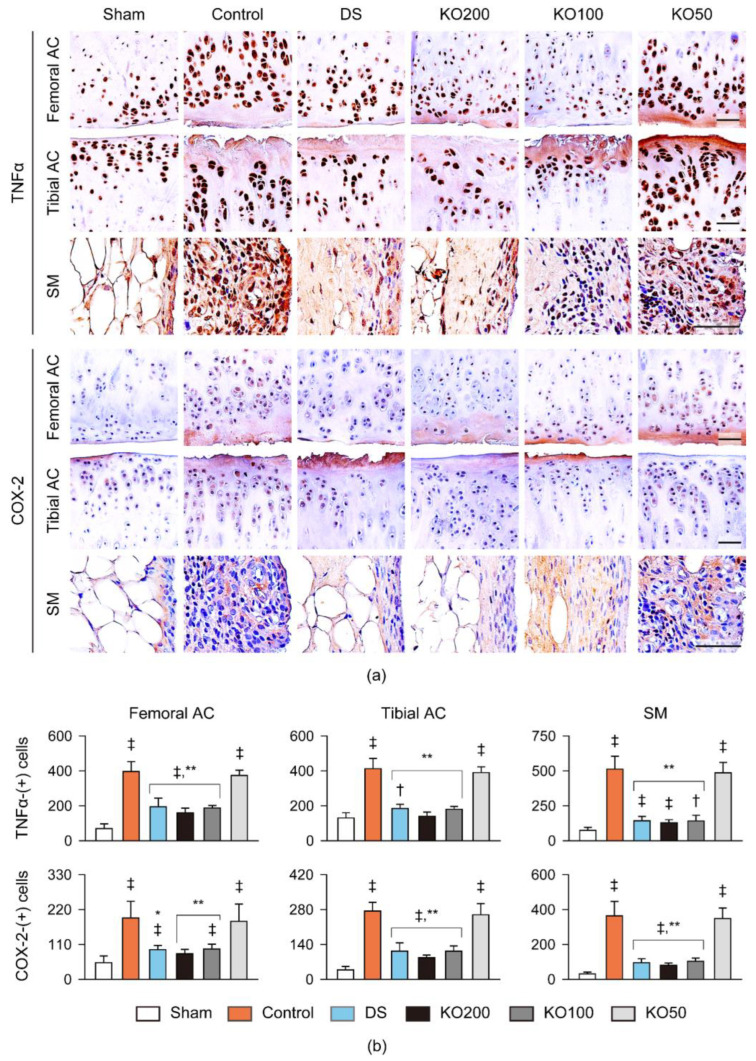
Immunohistochemistry for inflammation in vivo. (**a**) Representative images of immunostains for tumor necrosis factor (TNF)α and cyclooxygenase (COX)-2 in the femoral and tibial AC and the SM. Scale bars = 100 μm. (**b**) Immunoreactive cells for TNFα and COX-2 in both AC and the SM. Values are expressed as the means ± SDs in eight sample sizes. ‡: *p* < 0.01 and †: *p* < 0.05, compared to the sham; **: *p* < 0.01 and *: *p* < 0.05, compared to the control.

**Figure 10:**
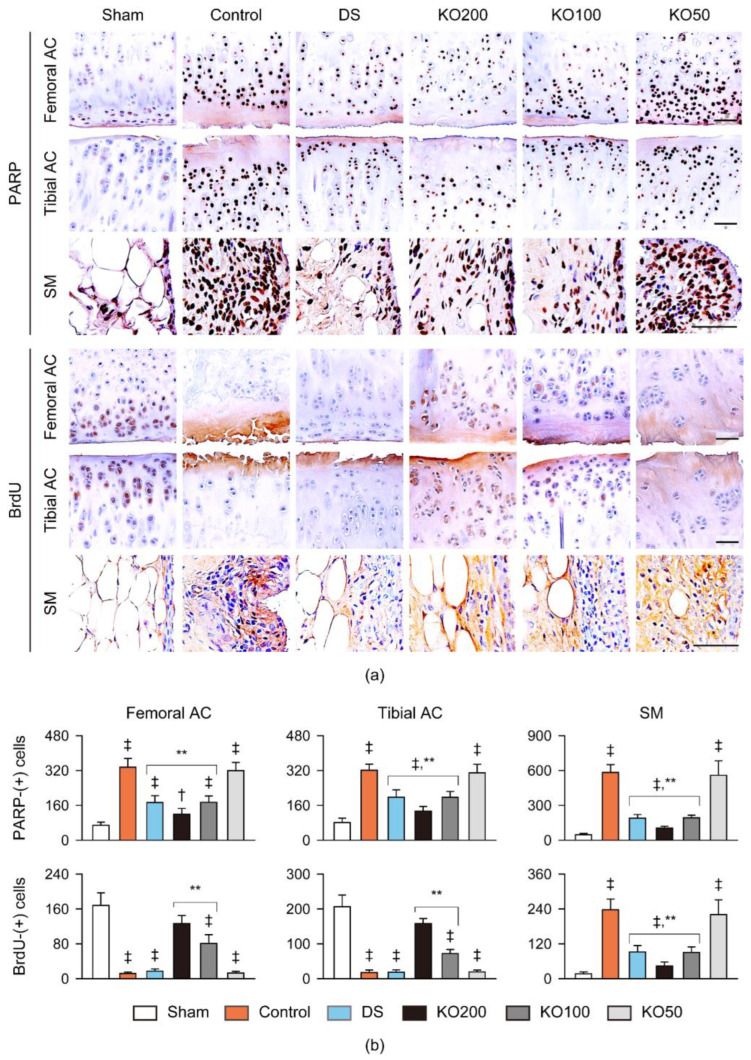
Immunohistochemistry for apoptosis and cell proliferation in vivo. (**a**) Representative images of immunostains for cleaved poly (ADP-ribose) polymerase (PARP) and bromodeoxyuridine (BrdU) in the femoral and tibial AC and the SM. Scale bars = 100 μm. (**b**) Immunoreactive cells for PARP and BrdU in both AC and the SM. Values are expressed as the means ± SDs in eight sample sizes. ‡: *p* < 0.01 and †: *p* < 0.05, compared to the sham; **: *p* < 0.01, compared to the control.

The femoral and tibial articular cartilages and the synovial membrane/infrapatellar fat pad were immunostained for tumor necrosis factor (TNF)α and cyclooxygenase (COX)-2 as markers of inflammation, cleaved poly (ADP-ribose) polymerase (PARP) as a marker of apoptosis, and bromodeoxyuridine (BrdU) as a marker of cell proliferation (Figure 9 and Figure 10). The immunostains for TNFα, COX-2, and PARP were evident in both articular cartilages and the synovial membrane/infrapatellar fat pad in the OA control; however, they were attenuated in the regions of the DS and KO treatments (Figure 9a and Figure 10a). The immunostain for BrdU differed between the articular cartilages and synovial membranes in the OA control, with little stains and pronounced stains, respectively (Figure 10a). However, the immunostains were reversed in the regions of the DS and KO treatment groups. The number of immunoreactive cells for TNFα, COX-2, and PARP were increased in both articular cartilages and the synovial membranes of the OA control compared to those of the sham (*p* < 0.01); however, they were significantly reduced in the regions of the DS, KO200, and KO100 groups compared to those of the OA control (*p* < 0.05, Figure 9b and Figure 10b). In particular, the PARP-immunoreactive cells were reduced more in the KO200 than in the DS (*p* < 0.01). The BrdU-immunoreactive cells were reduced in both articular cartilages of the OA control to the sham (*p* < 0.01); however, they were significantly increased only in the cartilages of the KO200 and KO100 compared to those of the OA control (*p* < 0.01, Figure 10b). Conversely, the immunoreactive cells in the synovial membranes were increased in the OA control to the sham (*p* < 0.01); however, they were significantly reduced in the DS, KO200, and KO100 compared to the OA control (*p* < 0.01).

## 3. Discussion

The knee OA rat model exhibited significant cartilage degradation, subchondral bone remodeling, and osteophyte formation, with synovitis-related edematous joint lesions, similar to previous studies [40,41]. However, the oral administration of KO at 200 and 100 mg/kg reduced 5-LO activity and PGE_2_ level in the articular cartilages and synovial membrane/infrapatellar fat pad, eventually leading to improvements in joint swelling and mobility. Additionally, the oral KO doses resulted in increased BMD and compressive strength in the cartilage, accompanied by the upregulation of chondrogenic genes (*Col-2*, *aggrecan*, and *Sox9*) and the inhibition of ECM degrading enzymes (MMP-2 and MMP-9). The stimulation of *Col-2* and *aggrecan* is known to provide bony tensile and compressive strength, respectively, as well as promotion of chondrocyte differentiation [42]. Furthermore, increases in the subchondral BMD and chondrogenesis are positively correlated with mechanical loading in knee OA [43]. It is speculated that KO may contribute to the formation of well-preserved cartilage tissues that serve as scaffolds for mineral deposition and enhance proliferative and anti-apoptotic activities in the knee joint. Consistently, in vitro KO treatments demonstrated the chondroprotective effects by increasing cell viability in chondrocytes exposed to IL1α, along with the upregulation of chondrogenic genes and the inhibition of ECM-degrading enzymes. The KO treatments also demonstrated the anti-inflammatory effects in the lipopolysaccharide-induced chondrocyte model. Histopathological and immunohistochemical analyses revealed that KO improved joint destruction and synovial inflammation, probably due to the anti-inflammatory, anti-apoptotic, and chondrogenic effects. These findings suggest the therapeutic potential of KO for knee OA.

Synovial levels of proinflammatory cytokines (i.e., TNFα and IL1) and systemic C-reactive protein (CRP), are found to be elevated in patients with knee OA [44,45], although the degree of inflammation is often modest [46]. The cartilage degradation triggers an inflammatory process in the localized joint, infrapatellar fat pad, and synovium, and the inflammatory mechanism is an important factor contributing to the structural damage and subsequent symptoms [4]. The inflammatory process initiates the infiltration of inflammatory cells into the synovium and increases the production of leukotrienes and PGE_2_ by stimulating the activities of 5-LO and COX-2, respectively, in coordination with proinflammatory cytokines such as TNF-α and IL1 [4,7]. Consequently, it leads to joint pain and progressive subchondral sclerosis over time. In this context, KO exhibited the capacity to suppress the inflammatory cascade both in vitro and in vivo, suggesting that its anti-inflammatory properties can provide symptomatic relief and potential improvement in the outcomes of knee OA. It has been reported that oral administration of KO or multi-ingredients consisting of KO (comprising 70% of the mixture), astaxanthin, and sodium hyaluronate inhibit OA progression through the nuclear factor-kappa B-mediated anti-inflammatory pathway in a MIA-induced knee OA rat model [47,48]. Previous studies also demonstrate that KO and n-3 PUFA, such as EPA and DHA, inhibit the production of prostaglandins, TNFα, and CRP [21], and improve the cartilage degradation and histopathological scores in OA animal models [22,23,47]. Additionally, the antioxidant astaxanthin is also known to inhibit the production of PGE_2_ and TNFα [28,29]. KO serves as a rich source of unique phospholipid carriers of n-3 PUFAs, EPA and DHA, and antioxidants. This suggests that the anti-OA effects of KO through anti-inflammatory mechanisms can be a promising priority in the treatment of knee OA.

Alongside impairments in de novo ECM synthesis, the inflammatory process inhibits the synthesis of ECM components such as Col-2 and proteoglycans in OA. This process further increases the production of the catabolic enzymes, exacerbating the destruction of cartilage ECM [49]. Progressive ECM degradation eventually leads to the formation of abnormal bone and painful osteophytes, resulting in cartilage destruction and the subsequent limitation of joint function [50]. The anatomical and physiological changes in OA could be triggered by metabolic and molecular alterations within the joint. MMP-induced ECM degradation is a major pathogenic factor responsible for cartilage degeneration in OA [6]. MMP-2 and MMP-9 exert catabolic actions to degrade collagen denatured by collagenases and cleaved aggrecan molecules [51]. Indeed, both MMPs and proinflammatory cytokines are highly expressed in the articular cartilages of OA patients, while their normal expression levels are lower [52]. *Sox9* is a chondrogenic transcription factor that regulates downstream genes such as *Col-2* and *aggrecan* as essential components of the ECM [53]. In the present study, KO treatments were observed to reverse the altered expressions of transcription factors, proteoglycans, and MMPs both in vitro and in vivo. Although *Col-2* was upregulated in the synovial membrane/infrapatellar fat pad of the OA model control, the expression was downregulated in the KO200 and KO100. This downregulation might contribute to the inhibition of joint fibrosis. The results of BrdU-positive cell proliferation might link to increased chondrocytes in the cartilages and reduced inflammatory cells in the synovial membranes, considering the chondrogenic and anti-inflammatory effects of KO, respectively. Moreover, KO demonstrated anti-apoptotic effects in the cartilages and synovial membranes, which could further improve the development of articular cartilage [38]. These findings provide valuable insights into the protective effects of KO on the pathological degeneration and physiological remodeling of cartilage tissue in knee OA.

Diclofenac is the most widely prescribed NSAID worldwide to relieve OA symptoms; however, long-term use also raises safety concerns due to gastrointestinal, cardiovascular, and renal adverse effects, similar to other NSAIDs [10,11]. The anti-OA effects of diclofenac sodium were significantly similar with the KO200 and KO100 groups; however, the effects on cell proliferation were not observed in the DS group, possibly due to its detrimental effects on bone regeneration by suppressing proteoglycan synthesis [12]. Furthermore, the KO200 group showed higher properties for the cartilage-protective effects (in gene expressions of aggrecan and Sox9, histopathological cartilage thickness and Mankin scores, and PARP-immunoreactive cells) and inhibitory effects on synovitis (in thickness of the synovial membrane-lining epithelium and 5-LO activity) compared to the DS group in the present in vivo study. Krill is the largest biomass in the world, with an estimated 300,000 million metric tons in the Antarctic Ocean [54]. Both KO and fish oils are important marine sources of the long-chain n-3 PUFAs; however, the phospholipid form of n-3 PUFAs in KO possesses higher bioavailability and absorption rates compared to the triglyceride form of n-3 PUFAs in fish oil [27,28]. Herein, no cytotoxicity was observed in KO treatments at up to 10 mg/mL in vitro and no abnormal effects on body weight or gait were noted with the oral administration of up to 200 mg/kg in vivo. The maximum oral dose of KO is approximately 2 g in an adult weighing 60 kg, calculated using the formula: 200 mg/kg (dose of KO) × 60 kg (body weight in adult) × 1/6 (a constant value for body surface area relative to human). Dietary supplementation of KO at 2 g/day for 30 days alleviated knee pain and stiffness in patients with chronic inflammation including OA [31], and the consumption of up to 4 g/day for 6 months improved the symptoms in patients with mild to moderate knee OA without the adverse effects [32]. Given the global burden of OA disease requiring long-term management, these results provide valuable information supporting that KO can be a promising alternative agent or functional food ingredient for the treatment of knee OA.

The anti-OA effects of KO were examined in a surgically induced OA animal model that reflects early post-traumatic knee OA with evident inflammatory responses [55]. While the current animal model offers the advantage of presenting reproducible disease progression, it cannot entirely replicate all stages of human knee OA, including the naturally occurring degenerative changes. For example, a high n-6/n-3 PUFA ratio is associated with joint pain and functional limitation in knee OA; however, lower proportions of n-6 PUFAs and reduced n-6/n-3 PUFA ratios have been reported in the synovial fluids of patients with the end-stage of the disease [56,57]. Moreover, anatomical and pathophysiological differences could result in inconsistent efficacy between clinical and preclinical studies. These disparities should be carefully considered in future clinical studies focusing on early OA in humans. Nevertheless, considering that clinical trials involving dietary KO supplementation have demonstrated beneficial effects in attenuating knee joint pain and improving joint motion, these findings suggest the relevant mechanisms of KO through anti-inflammation, anti-ECM degradation, anti-apoptosis, and chondrogenesis in the early stage of OA for the fundamental cure. Therefore, dietary supplementation with KO, a natural combination of EPA, DHA, and astaxanthin, may offer a safe and effective alternative treatment for knee OA.

## 4. Materials and Methods

### 4.1. Preparation of KO

The commercial product of Antarctic KO (Superba^TM^ Boost) was produced by Aker Biomarine (Houston, TX, USA) and purchased from SC Science (Ilsan, Republic of Korea). The KO was used in the same LOT number of products used in a previous study [39]. High-performance liquid chromatography analysis showed that the KO contained 51.2% (*wt.*/*wt.*) phospholipids, with 44.9% phosphatidylcholine, 3.6% 1-palmitoyl-2-hydroxyl-glycero-3-phosphocholine, 2.1% phosphatidylethanolamine, and 0.6% N-Acyl-phosphatidylethanolamine. The content of EPA and DHA in the KO was measured at 29.9% (*wt.*/*wt.*). The KO was dissolved in DW as a vehicle and stored at 4 °C until use.

### 4.2. Animals

All animals were handled in accordance with national regulations of the usage and welfare of laboratory animals, and the experimental protocols were approved by the Institutional Animal Care and Use Committee of Daegu Haany University (Approval No. DHU2021-069 and DHU2021-072 for in vivo and in vitro studies, respectively). The experiments adhered to the ARRIVE guidelines [58]. Six-week-old male specific pathogen-free (SPF)/viral antibody-free (VAF) Sprague Dawley rats were obtained from Orient Bio Inc. (Seongnam, Republic of Korea). They were housed in a temperature (20–25 °C)- and humidity (40–45%)-controlled room with a 12:12 h light-dark cycle. Feed and water were provided ad libitum. Following a two-week acclimatization period, the rats were anesthetized using a mixture of 70% N_2_O and 28.5% O_2_ with 2–3% isoflurane for induction and 1–1.5% isoflurane for maintenance, for knee joint sampling or surgical induction of the OA model. All animals were euthanized using CO_2_ gas.

### 4.3. Primary Culture of Rat Articular Chondrocyte and Treatment

Rat chondrocytes were isolated from knee cartilage, as described previously [59]. Briefly, cartilage was dissected from the subchondral bone and minced into approximately 1~3 mm^3^ pieces. The tissue was then digested using 0.2% pronase (Sigma-Aldrich, St Louis, MO, USA) for 0.5 h at 37 °C and 0.1% collagenase (Sigma-Aldrich) for 4 h at 37 °C. The chondrocytes were collected after brief centrifugation and cultured in Dulbecco’s modified Eagle’s medium supplemented with 100 U/mL penicillin, 100 mg/mL streptomycin, and 0.25 mg/mL amphotericin B (all from Life Technologies, Carlsbad, CA, USA), in a 37 °C humidified incubator with 5% CO_2_. The culture medium was changed every other day. Cells were passaged or seeded at 80% confluency and within five passages. They were seeded on a 24-well plate (2 × 10^4^ cells/well for cell viability and for 5-LO activity and PGE_2_ level; 5 × 10^4^ cells/well for MMP level; 5 × 10^6^ cells/well for gene expressions), and cultured overnight. The cells were treated with KO at five doses of 0.0001, 0.001, 0.01, 0.1, and 1 mg/mL in the culture medium containing 2% fetal bovine serum for 24 h. Cell injury and inflammation were induced by recombinant human IL1α at 5 ng/mL and LPS at 50 μg/mL, respectively [59]. The cell culture supernatants were collected for biochemical analyses of 5-LO, PGE_2_, and MMPs, and the cells were harvested for chondrogenic gene expression analysis.

### 4.4. Cell Viability Assay

Cell viability was assessed using the 3-(4,5-dimethylthiazol-2-yl)-2,5 diphenyl tetrazolium bromide (MTT) assay. Cells were incubated with 2.5 mg/mL MTT (Sigma-Aldrich) at 37 °C for 4 h, and the resulting formazan crystals were dissolved in dimethyl sulfoxide. Absorbance was measured at 570 nm with a reference wavelength of 650 nm using a microplate reader (Sunrise; Tecan, Männedorf, Switzerland). Cell viability was represented as a percentage relative to the vehicle-treated control.

### 4.5. Surgical Induction of OA and Treatments

The knee OA rat model was induced through antiterror cruciate ligament (ACL) transection and partial medial meniscectomy in the left knee, as described previously [40]. After exposing the medial joint capsule, the ACL was transected with partial removal of the anterior medial meniscus. The sham group underwent the same surgical procedure without the ACL transection and partial meniscectomy. Post-operative treatment was provided using povidone-iodine and Aluspray^TM^ (Vétoquinol, Paris, France). Two weeks after surgical induction, the OA model was regrouped into five groups based on body weight and knee thickness. The animals received treatments once a day for 8 weeks, either by oral administration of KO via a gastric gavage or subcutaneous injection of diclofenac sodium (Wako Pure Chemical Ind. Ltd., Osaka, Japan) in the dorsal back skin, in a volume of 5 mL/kg. The groups were as follows (*n* = 8 per group): sham and the OA model control orally with DW, the DS group subcutaneously with diclofenac sodium at 2 mg/kg in saline, or KO200, KO100, and KO50 groups orally with KO at 200, 100, and 50 mg/kg, respectively. The dose of diclofenac sodium and the maximum dose of KO were determined based on previous studies and clinical application of KO (equivalent to a human dose of 2 g in adults weighing 60 kg), respectively [39,40]. Animals were fasted overnight before OA induction, initial treatments, and euthanasia. Body weight and knee thickness were measured weekly. Three days prior to euthanasia, rats were intraperitoneally injected with BrdU (Sigma-Aldrich) at 50 mg/kg in saline.

### 4.6. Assessment of Joint Capsule Thickness and Extension Angle

The left hind limb from the coxofemoral to the ankle region was dissected carefully to preserve the joint capsule. The thickness of the joint capsule was measured using an electronic digital caliper (CD-15CPS, Mytutoyo, Tokyo, Japan), and the maximum angle of the joint extension was evaluated by a veterinarian blinded to the experimental groups [40].

### 4.7. Measurement of Focal BMD and Compressive Strength

Focal BMD and compressive strength were evaluated using DEXA (InAlyzer, MEDIKORS Inc., Seongnam, Republic of Korea) and a computerized testing machine equipped with a digital force gauge (JSV-H1000 and HF-10, Japan Instrumentation System Co., Tokyo, Japan), respectively, as described previously [39]. BMD was measured in the total and each region of the femoral and tibial articular cartilages along with their corresponding subchondral bones, by outlining boxes in the regions using image analysis software. The results were expressed in g/cm^2^. Compressive strength was assessed in the central region of the medial femoral and tibial condyles at a depth of 0.2 mm, expressed in Newton (N).

### 4.8. Assessment of 5-LO Activity and Levels of PGE2 and MMPs

The activity of 5-LO and levels of PGE_2_ and MMPs were evaluated in the chondrocyte culture supernatants and homogenates of joint tissue samples using the corresponding assay kits [40,59]. Tissue samples from the femoral and tibial cartilages and synovial membranes including infrapatellar fat pads were homogenized in RIPA solution using a bead beater (Taco^TM^Prep, GeneReach Biotechnology Corp., Taichung, Taiwan) and an ultrasonic cell disruptor (KS-750, Madell Technology Corp., Ontario, CA, USA). After centrifugation at 1200× *g* at 4 °C, the resulting supernatants were collected. The activity of 5-LO activity was assessed at 490 nm using a lipoxygenase inhibitor screening assay kit (#760700, Cayman Chemical, Ann Arbor, MI, USA). The level of PGE_2_ was measured at 450 nm using a PGE_2_ assay kit (#SKGE004B, Parameter^TM^, R&D Systems, Minneapolis, MN, USA), and the levels of MMP-2 and MMP-9 were determined at 560 nm using ELISA kits (#MBS494797 and #MBS722532, respectively, MyBioSource, San Diego, CA, USA).

### 4.9. Real-Time Reverse Transcription Polymerase Chain Reaction (RT-PCR)

Relative gene expressions of Col-2, aggrecan, and Sox9 were examined in cultured chondrocytes and joint tissue samples from the femoral and tibial cartilages and synovial membrane/infrapatellar fat pad, as described previously [40,59]. The RNA was extracted using TRIzol reagent (Invitrogen, Carlsbad, CA, USA), and its concentration and quality were assessed using the CFX96^TM^ Real-Time System (Bio-Rad, Hercules, CA, USA). Contaminating DNA was eliminated using the DNA-free DNA Removal kit (#AM1906, Thermo-Fisher Scientific, Rockford, IL, USA). The RNA was then reverse-transcribed with the High-capacity cDNA Reverse Transcription kit (#4368813, Thermo-Fisher Scientific), according to the manufacturer’s instructions. The cDNA products were combined with specific primers as follows: for *Col-2*, 5′-GAGTGGAAGAGCGGAGACTACTG-3′ and 5′-CTCCA TGTTGCAGAAGACTTTCA-3′; for *aggrecan*, 5′-GATGTCCCCTGCAATTACCA-3′ and 5′-TCTGTGCAAGTGA TTCGAGG-3′; and for *Sox9*, 5′-AGAGCGTTGCTCGGAACTGT-3′ and 5′-TCCTGGACCGAAAC TGGTAAA-3′, as forward and reverse oligonucleotides, respectively. The amplification was carried out under the following conditions—58 °C for 30 min; 94 °C for 2 min; 35 cycles of 94 °C for 15 s; 60 °C for 30 s; 68 °C for 1 min; and 72 °C for 5 min—using the ABI Step One Plus Sequence Detection system (Applied Biosystems, Foster City, CA, USA). The gene expressions were normalized to that of β-actin as an internal control gene using the comparative threshold cycle method. The primer for *β-actin* was forward 5′-ATCGTGGGCCGCCCTAGGCA-3′ and reverse 5′-TGGCCTTAGGGTT CAGAGGGG-3′.

### 4.10. Histopathological Analysis

The joint tissue samples were fixed in 10% neutral buffered formalin and then subjected to decalcification using a mixture of 24.4% formic acid and 0.5 N sodium hydroxide with daily exchanges for five days [40]. Following decalcification, the samples were longitudinally trimmed and embedded in paraffin. The paraffin-embedded samples were sectioned serially at a thickness of 3 μm, and the sections were stained with HE and Safranin O. Histopathological changes were examined for thicknesses of the articular cartilages and synovial membrane-lining epithelium, and the number of inflammatory cells in HE stains. The modified Mankin scores were assessed in Safranin O stains as the following four subgroups (0–3 points each); cartilage surface, hypocellularity, cell clustering, and stain intensity [40]. A higher score indicates a severe level of OA (semi-quantitative scores; max = 12). Histomorphometric analyses were conducted using a computer-assisted automated image analyzer (*i*Solution FL ver 9.1, IMT *i*-solution Inc., Vancouver, BC, Canada) by a histopathologist blinded to the experimental groups.

### 4.11. Immunohistochemistry

The other serial sections underwent pretreatment with trypsin (Sigma-Aldrich) and 2 N hydrochloric acid for antigen retrieval. Endogenous peroxidase activity was removed using 0.3% hydrogen peroxide and a non-specific binding protein was blocked with normal horse serum for 1 h. Then, the sections were incubated overnight at 4 °C with the following primary antibodies: mouse monoclonal antibodies for TNFα (#sc-52746, Santa Cruz Biotechnology, Santa Cruz, CA, USA; 1:200) and BrdU (#ab8152, Abcam, Cambridge, UK; 1:100), and rabbit polyclonal antibodies for COX-2 (#160126, Cayman; 1:200) and cleaved PARP (#9545, Cell Signaling Technology Inc., Danvers, MA, USA; 1:100). The next day, the sections were incubated with horse biotinylated secondary anti-mouse IgG or anti-rabbit IgG antibody for 1h, followed by ABC reagents (#PK-6200, Vector Lab., Burlingame, CA, USA) for 30 min. Immunoreactivity was visualized using a peroxidase substrate kit (Vector Lab.) and counterstained with hematoxylin. The staining process was conducted in a humidity chamber, and sections were rinsed with PBS three times between each step. Immunostains omitting primary antibodies were used as the negative controls. Cells occupying immunoreactive regions over 20% were counted by a histopathologist blinded to the experimental groups.

### 4.12. Statistical Analyses

The data are presented as means ± standard deviations in six independent experiments in vitro and eight sample sizes in vivo. The normal distribution of variables and homogeneity of variances were examined by the Kolmogorov–Smirnov and Levene tests, respectively. Given the normal distribution of the variables, the data were examined by one-way ANOVA. The kinetic changes of body weight and knee thickness were examined by two-way ANOVA with main factors for the groups and timepoints measured. The timepoints were treated as a repeated measure. Multiple comparisons were conducted using the Tukey HSD and Dunnett’s T3 post hoc tests for cases of equal and non-equal variances, respectively. A *p*-value less than 0.05 was considered statistically significant.

## Data Availability

Data are contained within the article.

## Abbreviations

5-LO: 5-lipoxygenase; AC: articular cartilage; ACL: antiterror cruciate ligament; ANOVA: analysis of variance; BMD: bone mineral density; BrdU: bromodeoxyuridine; Col-2: type 2 collagen; COX: cyclooxygenase; CRP: C-reactive protein; DEXA: dual-energy X-ray absorptiometry; DHA: docosahexaenoic acid; DW: distilled water; EC_50_: effective concentration producing the half maximal response; ECM: extracellular matrix; EPA: eicosapentaenoic acid; FA: fatty acid; HE: hematoxylin and eosin; IF: inflammatory; IL: interleukin; KO: krill oil; n-3: omega-3; LPS: lipopolysaccharides; MIA: monosodium iodoacetate; MMP: matrix metalloproteinase; MTT: 3-(4,5-dimethylthiazol-2-yl)-2,5 diphenyl tetrazolium bromide; NSAIDs: non-steroidal anti-inflammatory drugs; OA: osteoarthritis; PARP: poly (ADP-ribose) polymerase; PGE: prostaglandin E; PUFA: polyunsaturated FA; RT-PCR: reverse transcription polymerase chain reaction; SD: standard deviation; SM/IPFP: synovial membrane/infrapatellar fat pad; Sox9: sex-determining region Y-box 9; SPF: specific pathogen free; TNF: tumor necrosis factor; and VAF: viral antibody free.
